# A novel gene signature based on the hub genes of COVID-19 predicts the prognosis of idiopathic pulmonary fibrosis

**DOI:** 10.3389/fphar.2022.981604

**Published:** 2022-09-06

**Authors:** Run Guo, Yuefei Zhou, Fang Lin, Mengxing Li, Chunting Tan, Bo Xu

**Affiliations:** ^1^ Department of Respiratory Medicine, Beijing Friendship Hospital of Capital Medical University, Beijing, China; ^2^ Department of Orthopedics Medicine, The First Hospital of China Medical University, Shenyang, China

**Keywords:** COVID-19, idiopathic pulmonary fibrosis, gene signature, prognosis, GEO

## Abstract

**Background:** Increasing evidence has demonstrated that there was a strong correlation between COVID-19 and idiopathic pulmonary fibrosis (IPF). However, the studies are limited, and the real biological mechanisms behind the IPF progression were still uncleared.

**Methods:** GSE70866 and GSE 157103 datasets were downloaded. The weight gene co-expression network analysis (WGCNA) algorithms were conducted to identify the most correlated gene module with COVID-19. Then the genes were extracted to construct a risk signature in IPF patients by performing Univariate and Lasso Cox Regression analysis. Univariate and Multivariate Cox Regression analyses were used to identify the independent value for predicting the prognosis of IPF patients. What’s more, the Kyoto Encyclopedia of Genes and Genomes (KEGG), Gene Ontology (GO), and gene set enrichment analysis (GSEA) were conducted to unveil the potential biological pathways. CIBERSORT algorithms were performed to calculate the correlation between the risk score and immune cells infiltrating levels.

**Results:** Two hundred thirty three differentially expressed genes were calculated as the hub genes in COVID-19. Fourteen of these genes were identified as the prognostic differentially expressed genes in IPF. Three (MET, UCHL1, and IGF1) of the fourteen genes were chosen to construct the risk signature. The risk signature can greatly predict the prognosis of high-risk and low-risk groups based on the calculated risk score. The functional pathway enrichment analysis and immune infiltrating analysis showed that the risk signature may regulate the immune-related pathways and immune cells.

**Conclusion:** We identified prognostic differentially expressed hub genes related to COVID-19 in IPF. A risk signature was constructed based on those genes and showed great value for predicting the prognosis in IPF patients. What’s more, three genes in the risk signature may be clinically valuable as potential targets for treating IPF patients and IPF patients with COVID-19.

## Introduction

Idiopathic pulmonary fibrosis (IPF) is the most common form of chronic interstitial lung disease and accounts for 25%–30% of interstitial lung disease (Richeldi et al., 2014; Poletti et al., 2018). The primary symptoms of IPF are cough, breathlessness, restricted lung function, impaired gas exchange, and progressive lung damage (Martinez et al., 2017; Sgalla et al., 2018; Spagnolo et al., 2021). Studies have shown that the incidence, disability, and mortality rates of idiopathic pulmonary fibrosis are continuing to increase, with an annual incidence of between 3 and 18 per million in North America and Europe (American Thoracic Society, 1999; Ley and Collard, 2013; Hutchinson et al., 2015). Although there are differences in the pathogenesis of each individual with IPF, a growing number of studies have found that the prognosis for IPF was poor, with a median survival of only 3–5 years (Fernández Pérez et al., 2010; Esposito et al., 2015; Vancheri, 2012). Unfortunately, patients with IPF have limited treatment options, with lung transplantation being the only treatment effective. Although it can greatly improve patient survival, it is still challenging to universalize due to limited donors.

COVID-19 is an acute respiratory infectious disease caused by a novel coronavirus severe acute respiratory syndrome coronavirus 2 (SARS-CoV-2) (Acter et al., 2020). Patients infected with SARS-CoV-2 experienced severe lung infection and damage, which may lead to pulmonary fibrosis. COVID-19 and IPF share many symptoms, including shortness of breath, cough, breathlessness, as well as weakness, and muscle and joint pain (Wong and Tam, 2004; Shereen et al., 2020; Sohrabi et al., 2020; Martinez et al., 2021). Some studies have shown a subtle link between pulmonary fibrosis and COVID-19 (George et al., 2020; Sheng et al., 2020). In patients with IPF, a large amount of ACE2 accumulates in the airways, which triggers an acute cough that progresses to pneumonia. Similarly, the COVID-19 is covered with a spherical lipid bilayer, with a prominent rim and highly glycosylated stinger protein, which binds to ACE2 during viral infection, triggering a sustained release of ACE2 after the invasion of the cell and infection of the next target cells (Abd El-Aziz and Stockand, 2020). The imaging features of IPF and COVID-19 have some similarities while there are still some differences. The HRCT features of COVID-19-associated pulmonary fibrosis include thickened interlobular septa, architectural distortion, reticular opacities, and even traction bronchiectasis or honeycombing, which is similar to IPF. However, traction bronchiectasis and honeycombing are more common in IPF and thickened interlobular septa, and reticular opacities might be reduced in some patients with COVID-19 after discharge (Raghu et al., 2011; Hariri et al., 2020; Lu et al., 2020; Guarnera et al., 2021; Li et al., 2021a). And IPF was identified as one of the most serious risk factors for COVID-19 (Sheng et al., 2020). Patients with IPF from COVID-19 infection had a much worse prognosis and faced complex complications even after recovery from COVID-19 (King et al., 2014; George et al., 2020; Sun et al., 2020). Therefore, it is imperative to identify new prognostic markers as well as therapeutic targets for patients with IPF patients and IPF patients with COVID-19.

Our study was intended to explore the hub genes involved in COVD-19 as biomarkers correlated to risk stratification in IPF. Herein, we firstly identified the hub genes involved in the progression of COVID-19 by performing the WGCNA algorithm. Then we identified their prognostic values and expression levels of the hub genes related to COVID-19 in IPF. Differentially expressed prognostic genes related to COVID-19 were used to establish a prognostic risk signature in IPF. Further, differentially expressed genes (DEGs) were analyzed between high-risk and low-risk groups. Through computational analysis, we explored molecular mechanisms, expression regulation, and immune cell infiltration. The aim of this study was to provide an immunogenomic map of IPF and to identify key genes associated with survival as candidates for clinical biomarkers and potential targets of intervention for curing patients with IPF and IPF with COVID-19.

## Materials and methods

**FIGURE 1:**
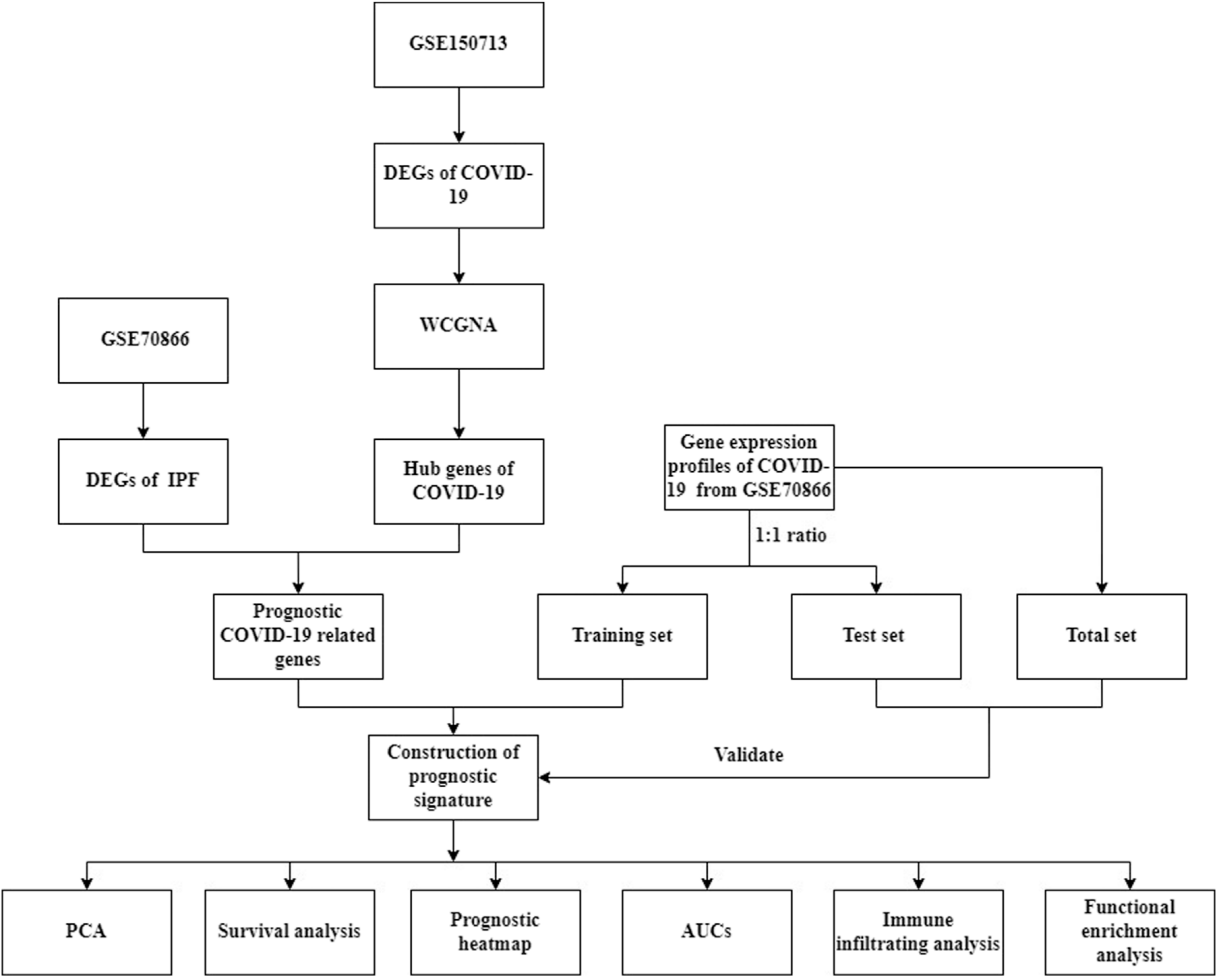
The flowchart of our research.

The flow chart of this research (Figure 1).

### Data collection

The expression profiles of blood samples in 100 COVID-19 patients and 26 non-COVID-19 individuals were extracted from the GSE157103 dataset on the GEO database (http://www.ncbi.nlm.nih.gov/geo/). Furthermore, we downloaded expression files of 196 bronchoalveolar lavages (BAL) samples (176 IPF samples and 20 normal samples) and related clinical information from the GSE70866 dataset. Patients with IPF were randomly divided into a training set (*n* = 88) and a test set (*n* = 88). There were no significant differences in analyzing the clinical variables between these two sets (Table 1).

**TABLE 1:** The clinical characteristics of different sets.

Characteristics	Test (*n* = 88)	Training (*n* = 88)	Total (*n* = 176)	*p*-Value
Age				1
<70	47 (53.40%)	46 (52.28%)	93 (52.84%)	
≥70	41 (46.60%)	42 (47.72%)	83 (47.16%)	
Sex				0.17
Female	12 (13.64%)	20 (22.72%)	32 (18.18%)	
Male	76 (86.36%)	68 (77.28%)	144 (81.82%)	

### WCGNA construction and COVID-19-related key module identification

We obtained the differentially expressed genes (DEGs) between COVID-19 and non-COVID-19 at log2 |FC| ≥ 1 and FDR < 0.05 by R package limma. The expression data of the DEGs were extracted from GSE157103. Some genes were excluded when the standard deviation was zero. R package WGCNA was used to eliminate the genes and samples which were the outlier after a scale-free co-expression network was constructed. Details are as follows: the person’s connection grids and normal linkage strategy were both performed for all pair-wise genes. Then, a weighted contiguousness lattice was built utilizing a power capability A_mn = |C_mn|^β (C_mn = Pearson’s relationship among’s Gene_m and Gene_n; A_mn = contiguousness between gene m and gene n). β was a delicate thresholding boundary that could delimit or strengthen for underscoring among genes and punish frail relationships. After picking the force of six, the nearness was changed into a topological cross-over grid (TOM), which could quantify the organization availability of a gene characterized as the amount of its contiguousness with any remaining genes for network gene proportion, and the comparing uniqueness (1-TOM) was determined. To characterize genes with comparative articulation profiles into gene modules, normal linkage progressive bunching was directed by the TOM-based disparity measure with a base size (gene bunch) of thirty for the genes dendrogram. *Moduleeigengenes* algorithm was used to identify the module eigengene values. *p*-Values < 0.05 and correlation coefficient > 0.3 (Sun et al., 2020; Cen et al., 2022) were considered statistically significant, and the modules being chosen were determined as COVID-19-related crucial modules.

### Identification of the hub genes in COVID-19 and the DEGs related to COVID-19 in IPF

We calculated the correlation between modules and genes to obtain the GS. Meanwhile, the correlation between module feature vectors and genes was also calculated to obtain MM. Based on the cut-off criteria (|MM| > 0.5 and |GS| > 0.1), 233 genes with high connectivity in the clinically significant module were identified as hub genes (Tang et al., 2018). DEGs of IPF were identified by the screening criterion at log2 |FC| ≥ 1 and FDR < 0.05. The common genes between COVID-19 and IPF were validated by intersecting COVID-19 hub genes and DEGs of IPF.

### Construction of a protein and protein interaction network

The STRING (http://www.string-db.org/) database was used to construct the PPI network and explore the interactions between these common genes.

### Construction of COVID-19-related prognostic signature

Patients with IPF were split up into a training set and a test set at a ratio of 1:1. We identified COVID-19-related prognostic DEGs in IPF and developed a prognostic risk signature. Then the predictive capability was validated in the test set, and total set. Univariate Cox proportional hazard regression was employed to confirm the COVID-19-related DEGs with prognostic values of overall survival. A cut-off *p*-value < 0.05 was set to prevent omissions. Afterward, the least absolute shrinkage and selection operator (LASSO) penalized Cox proportional hazards regression was employed to avoid overfitting and a prognostic signature was constructed with the R package glmnet (Wang et al., 2019). The final model was determined by the value of penalty parameters(λ) corresponding to the lowest partial likelihood of deviance. The formula used to calculate the risk score was as follows: risk score = sum (COVID-19-related prognostic DEGs expression level × corresponding coefficient). Subsequently, patients were divided into high-risk and low-risk groups according to the median risk score. Principal component analysis was employed to implement by using R package stats. Predictive power was further validated by Kaplan–Meier survival curves and the area under the curves (AUCs) by conducting the R packages survival and survivalROC (Lorent et al., 2014), respectively.

### Functional enrichment analysis

All samples in GSE70866 were divided into high-risk and low-risk groups according to the prognostic risk score. Kyoto Encyclopedia of Genes and Genomes (KEGG) enrichment analysis and Gene Ontology (GO) were performed between high-risk and low-risk groups by employing the R package clusterProfiler using the DEGs (log2|FC| ≥ 1 and FDR < 0.05). Furthermore, Gene Set Enrichment Analysis (GSEA) was also conducted in the Hallmark gene set *h. all.v7.4. symbols.gmt* between high-risk and low-risk groups to explore the key biological pathways of DEGs by GSEA (version 4.1.0). An adjusted *p*-value < 0.05 was considered statistically significant.

### Immune infiltrating cells analysis

As a deconvolution algorithm based on RNA-Seq data, CIBERSORT can estimate the composition ratio of different immune cells (Newman et al., 2015). We then used the R package IOBR to calculate the relative proportions of twenty-one immune infiltrating cells in training cohort according to the transcriptional data. Wilcoxon rank-sum test was used to identify the differences in the immune cell infiltration in high-risk and low-risk groups. *p*-Value < 0.05 was considered statistically significant. What’s more, Pearson’s correlation analysis was conducted to calculate the correlation between hub genes and different immune infiltrating cells.

## Results

### Construction of WGCNA and identification of COVID-19-related key module

**FIGURE 2:**
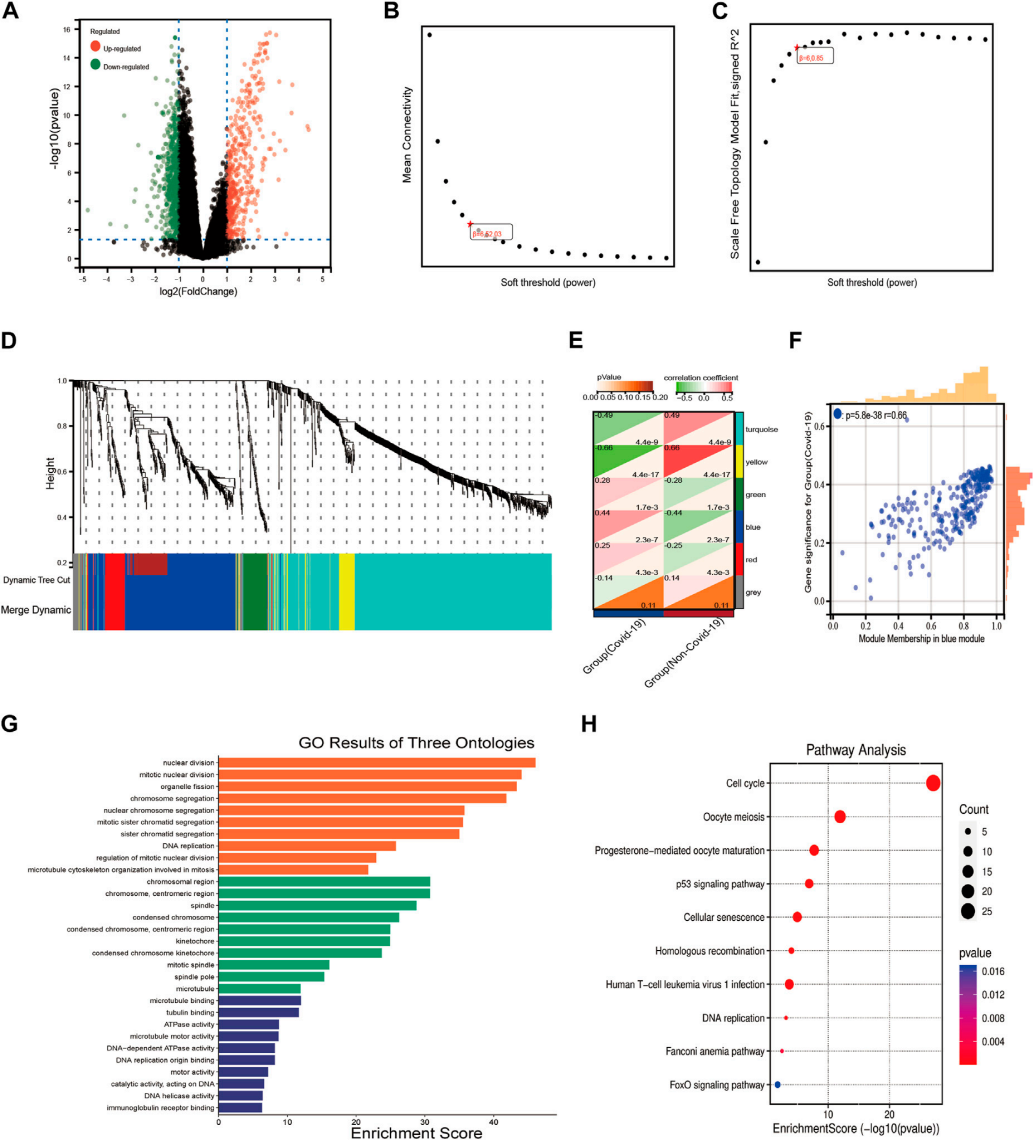
WGCNA analysis and the functional pathway analysis. **(A)** The results of the DEGs of COVID-19. **(B,C)** Network topology analysis of different soft threshold power. **(D)** Dendrograms of genes acquired by mean linkage hierarchical clustering, A color branch of the cluster tree represents a co-expression module. **(E)** Module-trait relationships. The red color represents positive correlation and the green color represents negative correlation. The significance of the correlation was indicated by *p*-value. **(F)** The correlation between blue module and COVID-19. **(G,H)** The results of GO and KEGG pathway enrichment analysis.

The differentially expressed genes in COVID-19 and normal individuals were identified, including 429 up-regulated genes and 647 downregulated genes (Figure 2A), then the expression profiles of these DEGs were extracted from the GSE157103 dataset. Further, we used WGCNA to analyze the expression profiles of the DEGs to find co-expressed gene modules. The scale-free topological index was 0.85 when the soft threshold for COVID-19 was six, as demonstrated in Figures 2B,C, therefore the network follows a power-law biological network, and the derived gene dendrograms and their corresponding module color was shown in Figures 2D,E. The correlations between each module and the two phenotypes (health and disease states) were determined by using hierarchical clustering and Spearman correlation analyses, and the modules most relevant to the disease were identified. The heatmap showed that the blue (r = 0.44, *p* = 2.3e-7) module was highly correlated to COVID-19 (Figure 2H). Meanwhile, the correlation between gene signatures (GS) for COVID-19 and module membership (MM) in the blue module (r = 0.66, *p* = 5.8e-38) was also validated the blue module was the key module associated with the progression of COVID-19. After that, 233 key genes in the blue module were extracted at the threshold of GS > 0.1 and MM > 0.5. the results of the GO and KEGG pathway enrichment demonstrated that these 233 genes mainly enriched in the biological pathways involved in cell proliferation, immunomodulation, and energy metabolism, including DNA replication, nuclear division, p53 signaling pathway, cell cycle, ATPase activity, and immunoglobulin binding.

### Identification of COVID-19-related prognostic DEGs in IPF

**FIGURE 3:**
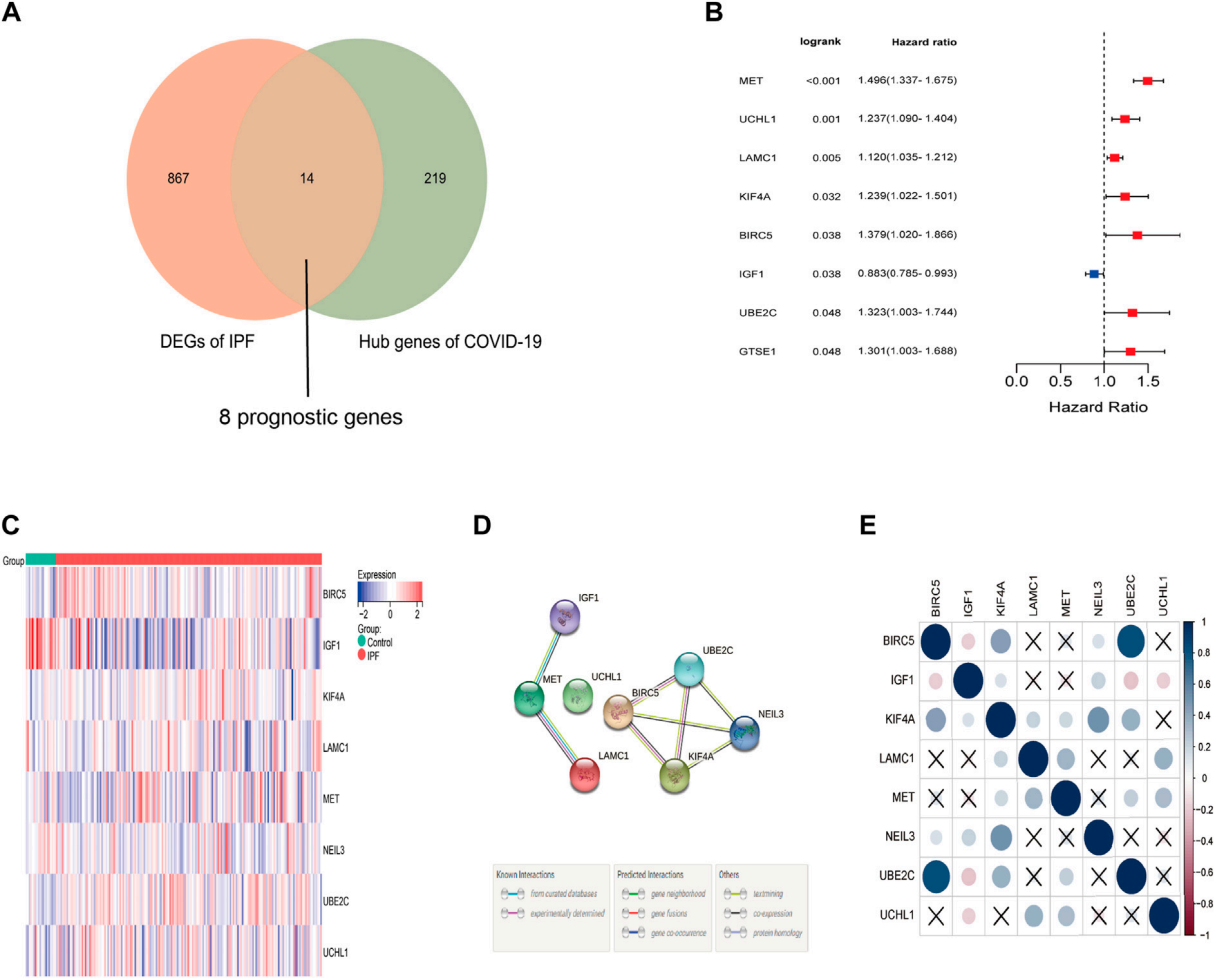
Identification of the prognostic DEGs related to COVID-19 in IPF. **(A)** Eight prognostic genes related to COVID-19 were differentially expressed between IPF group and normal group. **(B)** Univariate Cox proportional regression analysis showed that eight differentially expressed prognostic genes related to COVID-19 were significantly correlated to the survival of IPF patients. **(C)** The heat map of eight differentially expressed prognostic genes related to COVID-19. Red represents high expression and blue represents low expression. **(D)** The protein and protein network of eight differentially expressed prognostic genes related to COVID-19. **(E)** The correlation heat map of eight prognostic differentially expressed genes related to COVID-19.

Based on the log2 |FC| ≥ 1 and FDR < 0.5, a total of 14 differentially expressed genes were identified from the 233 COVID-19-related genes in IPF samples. Among which were thirteen up-regulated genes (BIRC5, UBE2C, GTSE1, KIF4A, CDC25C, BHLHA15, UCHL1, PIMREG, MET, CENPI, ERCC6L, LAMC1, NEIL3) and one downregulated gene (IGF1). What’s more, eight of these thirteen genes were correlated to the overall survival in the Univariate Cox regression (Figures 3A,B), and the heat map showed that the expression difference between IPF samples and matched normal samples (Figure 3C). The protein and protein interaction network and correlation heat map exhibited the interactive information among these differentially expressed prognostic genes related to COVID-19 (Figures 3D,E).

### Establishment of a risk signature related to COVID-19 in IPF

**FIGURE 4:**
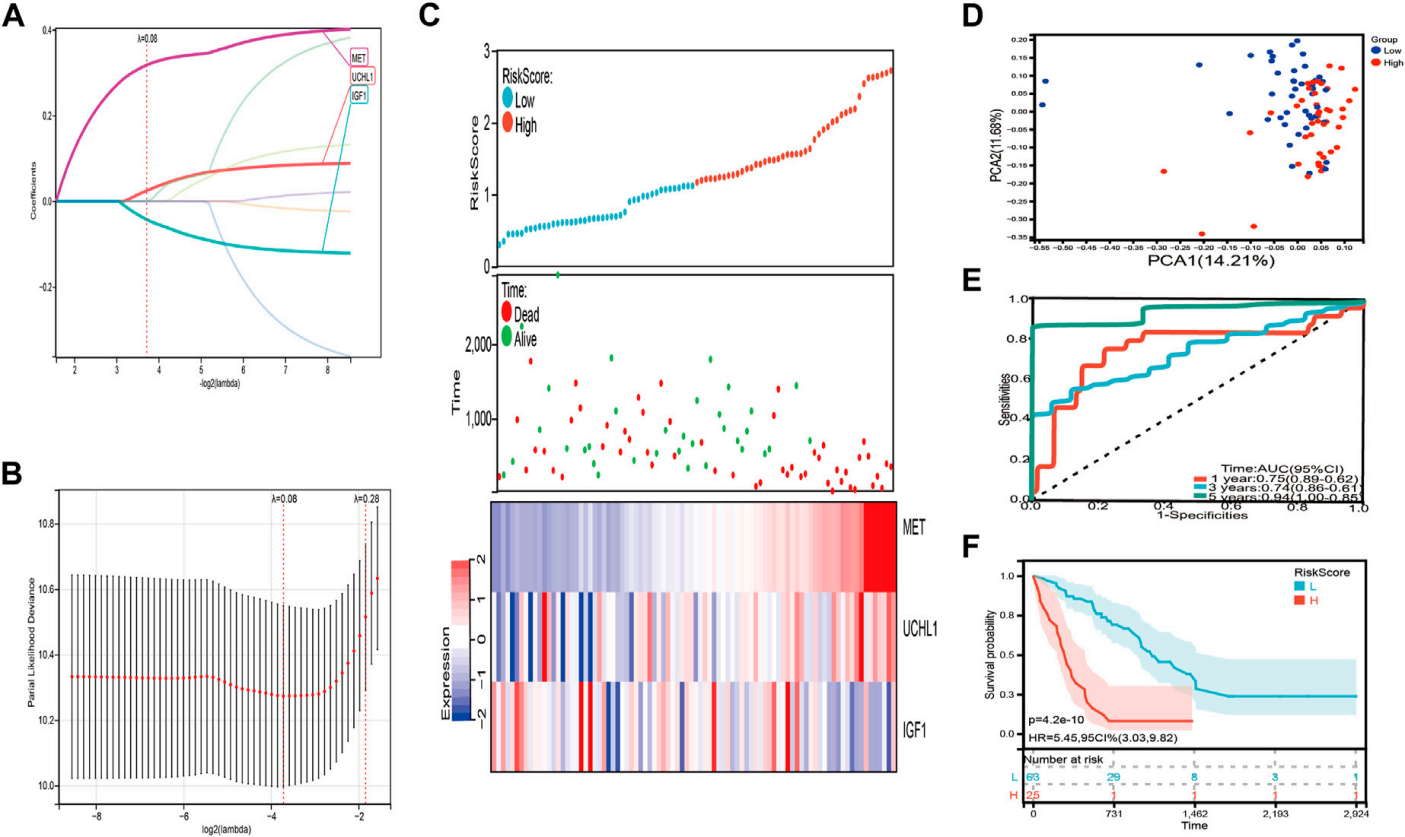
Construction of the risk signature in the training set. **(A,B)** Cross validation for tuning parameter selection and lasso analysis of eight differentially expressed prognostic genes related to COVID-19. **(C)** The distribution of risk score, survival status, and expression level of three differentially expressed prognostic genes related to COVID-19 between high-risk and low-risk group. **(D)** The PCA plot of the training set. **(E)** Time-dependent ROC analysis. **(F)** The overall survival of IPF patients in high-risk and low-risk group.

We used the eight prognostic genes related to COVID-19 to establish the risk signature in the training set, including seven detrimental genes with HR > 1 and one protective gene with HR < 1. Lasso Cox regression was performed to minimize the overfitting, three of the eight genes were chosen to construct a risk signature according to the optimum λ value (λ = 0.8) (Figures 4A,B). The risk score was calculated as the following formula: Risk score = 0.32 × MET + 0.0244 × UCHL1-0.0429 × IGF1. Subsequently, all patients with IPF in the training set were divided into high-risk and low-risk groups based on the median risk score. PCA analysis illustrated those patients with IPF were distributed in two directions (Figure 4D). We graded the risk scores of the IPF patients and depicted their distributions in the training set, the dot plot demonstrated the survival status of these patients, and the expression differences of the three prognostic genes were shown between two risk groups (Figure 4C). Patients with higher risk scores had a considerably lower overall survival (*p* = 4.2e-10, HR = 5.45, 95 CI% = 3.03–9.82) (Figure 4F). The areas under the curve (AUCs) were 0.75 at 1 year, 0.74 at 3 years, and 0.94 at 5 years (Figure 4E).

**FIGURE 5:**
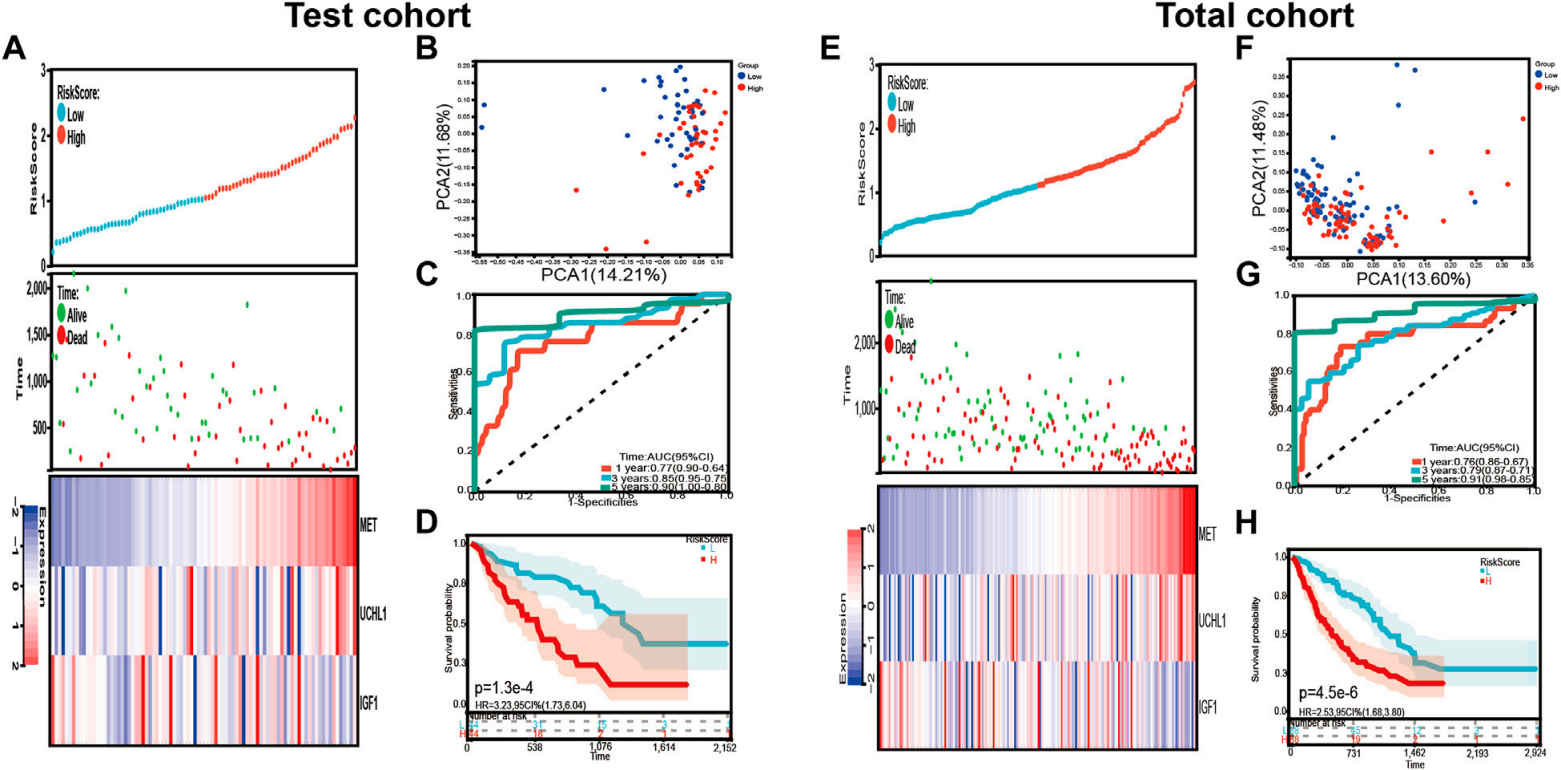
Validation of the risk signature in the test cohort and total cohort. **(A,E)** the distribution of risk score, survival status, and expression levels of three differentially expressed prognostic genes related to COVID-19 in test cohort and total cohort. **(B,F)** The PCA plot of test cohort and total cohort **(C,G)** Time-dependent ROC analysis of our risk signature in test cohort and total cohort. **(D,H)** The overall survival of IPF patients in test cohort and total cohort.

In addition, the risk signature’s predictive potential was validated in both the test and total set. Every patient’s risk score was determined, and the patients were separated into high-risk and low-risk groups in two sets, as previously reported. Figure 5A presented the distribution of the risk scores, survival status, and the expression level of the three prognostic genes related to COVID-19. The result of PCA revealed that the patients in each subgroup were divided into two clusters (Figures 5B,F). The Kaplan–Meier survival curves in the test group showed that the overall survival of high-risk patients was lower than that of the low-risk groups (*p* = 1.3e-4, HR = 3.23, 95% CI = 1.73–6.04) (Figure 5D). The AUC after 1 year was 0.77, the AUC after 3 years was 0.85, and the AUC after 5 years was 0.90 (Figure 5C).

Similar to the test cohort and the training cohort, the overall survival was subsequently different in the two risk groups (*p* = 4.5e-6, HR = 2.53, 95 CI% = 1.68–3.80) (Figure 5H). The AUC after 1 year was 0.76, the AUC after 3 years was 0.79, and the AUC after 5 years was 0.91 (Figure 5G). The distribution of the risk scores, survival status, and the expression levels of the three prognostic genes related to COVID-19 were also shown in Figure 5E.

### Independent prognostic analyses of the risk signature

**FIGURE 6:**
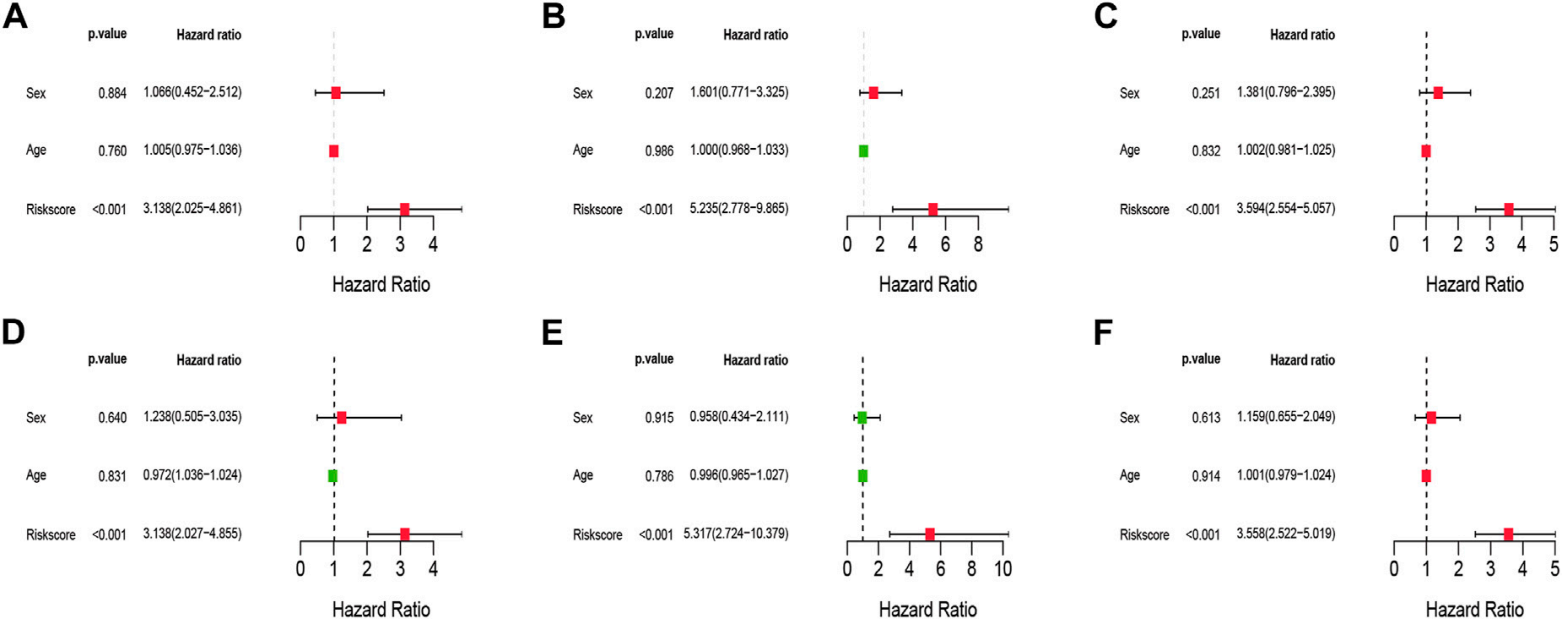
Univariate and Multivariate Cox analysis of the risk signature. **(A–C)** The Univariate Cox analysis of the risk signature in training cohort, test cohort, and total cohort. **(D–F)** The Multivariate Cox analysis of the risk signature in training cohort, test cohort, and total cohort.

To examine if clinical characteristics (such as gender and age) and risk score are independent prognostic variables for overall survival in IPF, Univariate and Multivariate Cox regression analyses were undertaken. The results showed that the risk score was remaining as an independent risk factor for the outcome of IPF in both training (Figures 6A,D), test (Figures 6B,E), and the total set (Figures 6C,F) after considering age and gender.

### Functional enrichment analyses based on the risk signature

**FIGURE 7:**
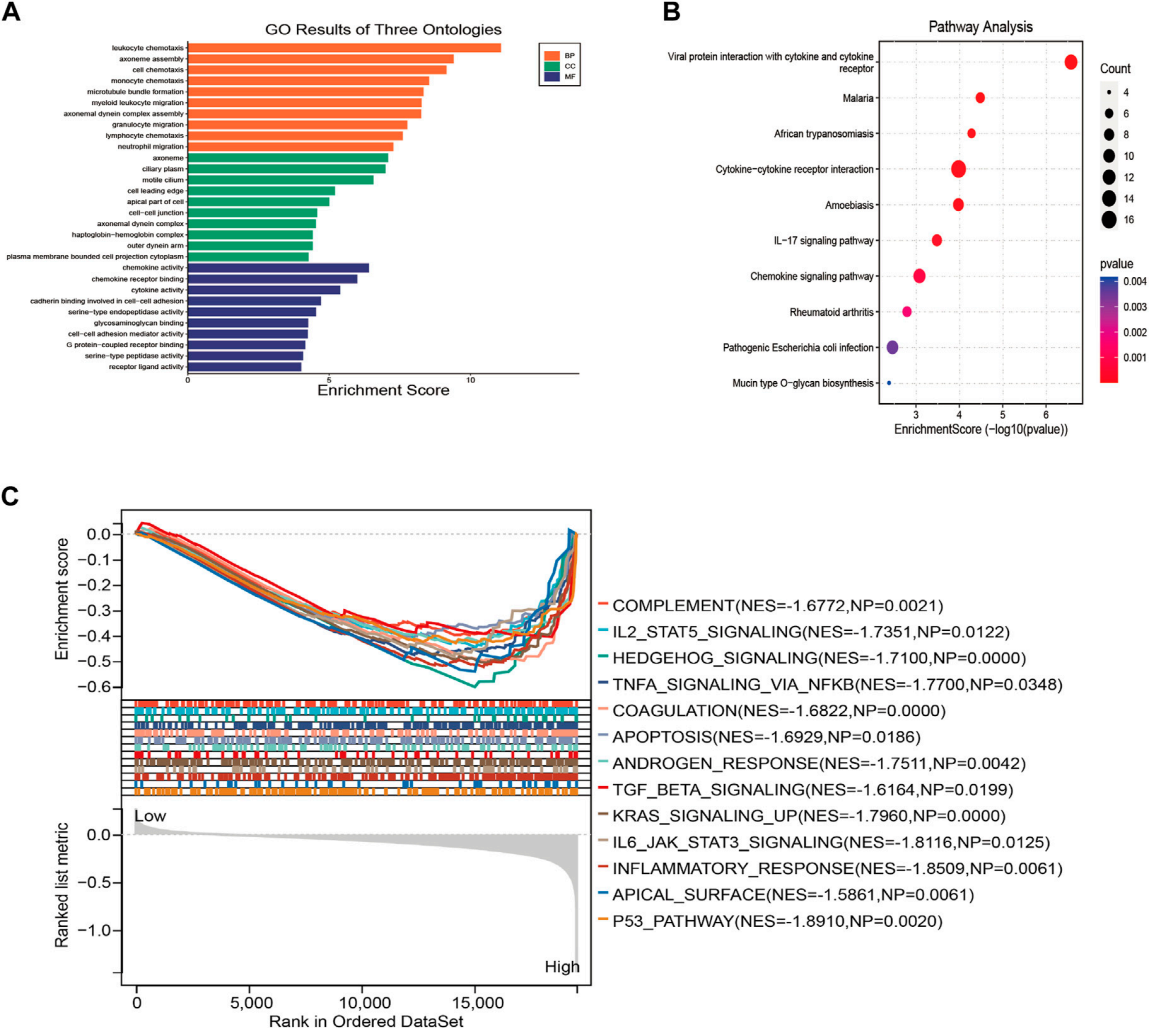
Functional enrichment analysis based on the DEGs from high-risk and low-risk groups in training cohort. **(A)** The results of GO pathway enrichment analysis. **(B)** The results of KEGG pathway analysis. **(C)** The enriched hallmarks of GSEA results based of the risk signature.

Three hundred ninty nine DEGs correlated with the risk score were identified in the training group at the criterion of log2 |FC| ≥ 1 and FDR < 0.5, GO and KEGG pathway enrichment analyses were performed on those DEGs to further elucidate the potential biological functions and pathways related to the risk score. The results showed that the DEGs were mostly enriched in cell chemotaxis, chemokine and cytokine activity, viral protein interaction with cytokine and cytokine receptors, and chemokine signaling pathway (Figures 7A,B).

### GSEA of the risk signature correlated to the COVID-19

GSEA was conducted to further investigate the function of the risk signature related to COVID-19, based on the expression matrix of all genes between the high-risk and low-risk groups. The results depicted that most genes in risk signature related to COVID-19 regulate the immune and detrimental hallmarks of IPF. Considerably, the biological pathways such as IL2-STAT5 signaling, apoptosis, TGFβ signaling, and inflammatory response were identified to be mainly enriched in the high-risk group (Figure 7C).

### Differences in the immune cell infiltrating between high-risk and low-risk groups

**FIGURE 8:**
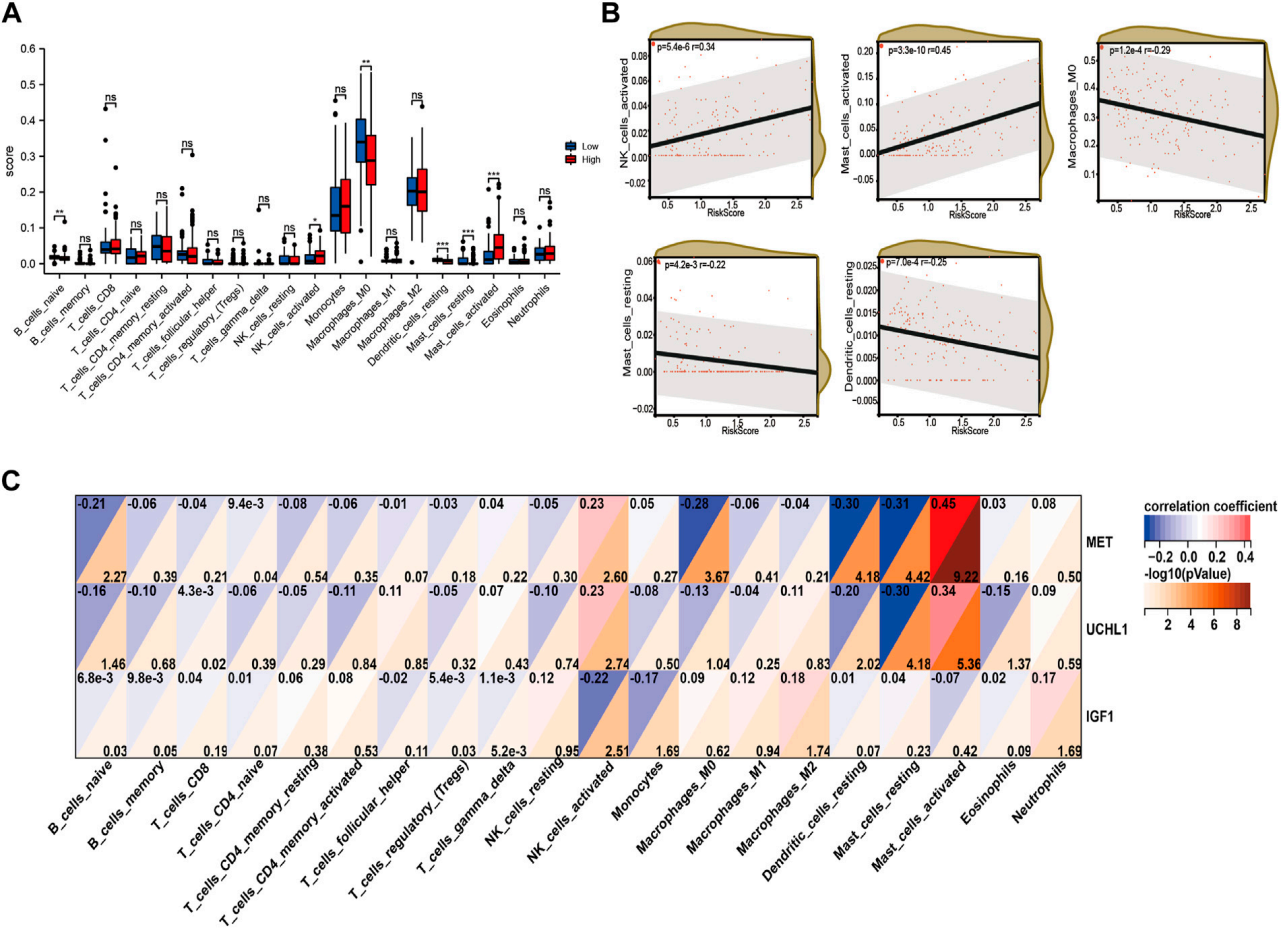
The immune infiltrating cell analysis based on the risk score in training cohort. **(A)** The infiltrating levels of twenty-three cells in high-risk and low-risk group. **(B)** Association between the infiltrating levels and risk score. **(C)** Correlation between MET, UCHL1, IGF1, and infiltrated immune cells.

Immune cells play a significant role in disease progression. Hence, we analyzed the immune infiltrating cells in the high-risk and low-risk groups to estimate the association between the risk signature we constructed and the immunoregulation. We utilized the CIBERSORT algorithm to calculate the relative ratio of the twenty-one immune cells in every patient with IPF. The results showed that the naïve B cells, M0 macrophages, resting dendritic cells, and resting mast cells were infiltrating more in the low-risk group, while activated NK cells and activated mast cells were infiltrating more in the high-risk group (Figure 8A). What’s more, the correlation analysis between risk score and immune cell infiltrating also demonstrated that the activated NK cells (*p* = 5.4e-6, r = 0.34) and activated mast cells (*p* = 3.3e-10, r = 0.45) were positively related to the risk score, while M0 macrophages (*p* = 1.2e-4, r = −0.29), resting mast cells (*p* = 4.2e-3, r = −0.22), and dendritic cells (*p* = 7.0e-4, r = −0.25) were negatively correlated to the risk score (Figure 8B). In addition, the expression levels of MET and UCHL1 were positively correlated to the activated mast cells and activated NK cells. However, the resting mast cells, resting dendritic cells, and M0 macrophages were negatively associated with both two genes. Furthermore, IGF1 was negatively correlated to the activated NK cells and monocytes.

## Discussion

As previously reported, courses of different IPF patients were hard to predict, some patients with IPF deteriorated rapidly while some others progressed much slowly (Ley et al., 2011; Mura et al., 2012; Mishra and Sindhwani, 2021). Meanwhile, treatments for patients with IPF were limited. When COVID-19 first came out in 2019, people found there was a strong link between IPF and COVID-19 (Uzel et al., 2020; Zhang C. et al., 2021). As a study showed that fifty-gene profiles in peripheral blood can predict the outcomes of IPF and COVID-19 patients (Juan Guardela et al., 2021). We supposed that interaction between IPF and COVID-19 may be through some key genes, so the present study was intended to explore some hub genes both involved in IPF and COVID-19.

In our research, we found three genes both involved a lot in IPF and COVID-19, the gene signature of these three genes can well predict the outcomes of IPF patients. The mechanisms behind the gene signature were further illustrated. MET, also called mesenchymal-epithelial transformation factor, was firstly validated in tumor progression (Tanaka et al., 2006; Watson et al., 2006). Three types of mutation in MET can lead to the coincidence of tumors (Paik et al., 2015; Skead and Govender, 2015). Its high expression indicated a poorer prognosis for IPF patients in our study. IPF has many resemblances with cancer, like invasive phenotype. Recently it was reported that ASS1 deficiency happened in pulmonary fibrosis, MET can be activated by knocking down ASS1, then interacting with the upstream of the Src-STAT3 signaling to up-regulate the proliferation of fibroblast cells (Li et al., 2021b). Moreover, it has been shown that the overexpression of the MET and CD44v6 can sustain the TGFβ signaling in IPF (Stella et al., 2014). MET blockage can lead to recovery from the damage of aberrant recapitulation of developmental programs. A study even proved that MET inhibitors can be tested in interfering with the progression of IPF (Stella et al., 2016). UCHL1, also called ubiquitin carboxyl-terminal hydrolase 1, was functionally identified to regulate a range of cellular processes, such as cell-cell communication, apoptosis, and DNA repair, by removing or editing poly or monoubiquitin chains from ubiquitin proteins (Clague et al., 2012; Pfoh et al., 2015). Its role in fibrosis was still lacking exploration, a study demonstrated it can be a potential therapeutic target for liver fibrosis (Wilson et al., 2015). More recently, there was a study showed that potent and selective UCHL1 inhibitors can down-regulate the fibrotic responses in a cellular model of IPF, which indicated that UCHL1 may be a potential target to cure IPF (Panyain et al., 2020). These results from others were consistent with our exploration of which UCHL1 acted as a detrimental gene in IPF. For insulin-like growth factor 1(IGF1), was mainly studied in various tumors. Several studies identified that IGF1 was involved in fibrotic progression and may play a great role in IPF (Salonen et al., 2022; Thomas et al., 2022). In our study, the expression level of IGF1 was lower in the IPF group and IGF1 was identified as a protective gene when its high expression predicted better survival of IPF patients. However, these three genes were seldomly studied in COVID-19 because of the explosive growth of COVD-19 and the risk to perform experiments. Future biological pathways behind COVID-19 will be revealed eventually.

The WGCNA algorithm was performed to investigate the key genes and possible pathways associated with COVID-19. Two hundred thirty three key genes were identified in the most crucial molecule. The results of KEGG and GO pathway enrichment indicated that those genes were mostly enriched in DNA replication, immunomodulation, and energy metabolism. Increasing studies of COVID-19 patients showed that SARS-Cov-2 infects and replicates in endothelial cells in multiple organs, such as the heart, lung, kidney, and liver (Dashtban et al., 2022; Giovannini et al., 2022; John et al., 2022; Kumar et al., 2022; Schold et al., 2022; Zheng et al., 2022). In addition, the enriched pathway p53 signaling was identified as a key pathway of genomic stability and cell cycle progression, it even plays a great part in the suppression of viral replication (Kumar et al., 2022). Those augments further explained the genes we explored were hub genes involved in the pathological course of COVID-19. These hub genes of COVID-19 further were further analyzed in the patients with IPF, eight of them were identified as the differentially expressed prognostic genes in IPF. Finally, we constructed a risk signature in three genes (MET, UCHL1, IGF1) correlated to COVID-19 in IPF by conducting Lasso Cox Regression analyses. The K-M and Roc analyses both revealed that the risk signature can well predict the survival of the IPF patients at 1-, 2-, and 5-years. Subsequently, the prognostic value of the risk signature we constructed was evaluated in our test set and total set, the results both demonstrated the risk signature was stable and considerable. Furthermore, the expression levels of those three genes were validated in our clinical samples. Univariate and multivariate cox regression was performed to validate the risk score was an independent risk factor by combining age and gender. More clinical information on IPF patients, such as their grades, smoking frequency, and treatments research should be collected to test this risk signature.

The results of functional enrichment analyses demonstrated that the DEGs between high-risk and low-risk groups were mainly enriched in immune-related pathways, such as monocyte chemotaxis, lymphocyte chemotaxis, chemokine receptor binding, chemokine activity, cytokine, cytokine receptor interaction, and IL-17 signaling pathway. The results of GSEA also indicated that the immune-related pathways were activated in high-risk groups, like TNFβ signaling, IL2-STAT5 signaling, TNFα signaling *via* NFκB, and inflammatory response. C-MET was reported that can increase the invasiveness of monocytes and play a role in regulating monocyte-macrophage function (Beilmann et al., 2000; Galimi et al., 2001). What’s more, a study showed that TGF-beta-1-2-, as well as IFN-beta,a induce HGF secretion by microglia and that antibodies to the HGF receptor c-Met abrogate OPC chemotaxis induced by TGF-beta2-treated microglia (Lalive et al., 2005). Neither mAb 10G10, which recognizes an epitope distinct from the one recognized by mAb 4B2, nor mAb UCHL-1, a CD45RO-specific antibody, induced any significant increase in TNF-alpha transcription (Estoppey et al., 1996). Inhibiting the classical NF-κB signaling pathway blocks growth hormone (GH) or insulin-like growth factor (IGF-1) signaling, suppresses cell proliferation, and suppresses bone morphogenetic protein 2 (BMP2) expression, thereby promoting apoptosis (Jimi et al., 2019). Most importantly, these three genes were all involved a lot in the inflammatory response (Zhang Z. et al., 2021; Zhu et al., 2021; Li et al., 2022), stating that inflammatory response was a main regulated pathway in IPF and these three genes could modulate the pathological process of IPF through this pathway.

Therefore, we speculated that these key genes from COVID-19 can also regulate the immune response in IPF patients. Moreover, we found that there were great differences in immune cell infiltrating between the high-risk and low-risk groups (naïve B cells, M0 macrophages, resting dendritic cells, resting mast cells, activated NK cells, and activated mast cells). What’s more, the correlation between those three genes and different immune cells was also validated. IPF was first depicted as a lung disease caused by inflammation (2000). Then the clinical trials for immunosuppressive agents in IPF showed no benefit, some were even indicated to be harmful (Izumi et al., 2012). In a murine model of fibrosis, alveolar macrophages from monocytes showed expansion by expression of profibrotic genes in lineage-tracing experiments. And if this line of macrophages was deleted, the fibrosis would be attenuated (Misharin et al., 2017; McCubbrey et al., 2018). Macrophages release multiple cytokines during lung injury, such as IL-6, IL-1, TNFα, and TNFβ, which can regulate epithelial cell proliferation. It also reported that macrophages may secret MMPs inhibitors to restrict fibrosis (Wick et al., 2013). Furthermore, it was reported that increased monocyte count and red cell width may present negative prognostic biomarkers in patients with IPF (Karampitsakos et al., 2021). Those results suggested that macrophages and monocytes play a causal role in IPF. For mast cells, there was a study showed that the density of mast cells related to many clinical characteristics of IPF. IPF patients would experience an acute deterioration when mast cells significantly decrease (Salonen et al., 2022). NK cells were also identified to involve in the progression of IPF in a cell analysis according to single-cell transcriptome data (Cruz et al., 2021). The activity and proportion of NK cells in lung tissue from IPF patients were decreased (Huang et al., 2021). So these three genes were speculated to regulate the immune response in IPF through immune cells and immune-related pathways.

The present study has some limitations. Firstly, transcriptome analysis does not reflect changes in overall immune status. Secondly, the sample size was too small to provide complete clinical information. Third, because the database provides limited information on clinical characteristics, some important factors, such as typing and grading, were not included in our analysis. Fourth, we didn’t perform cell and sample experiments *in vitro* and vivo. Therefore, very careful extrapolations based on these findings must be made.

Taken together, our study initially explored the key genes involved in the progression of COVID-19 and IPF. Then a risk signature was constructed in IPF based on the genes chosen from COVID-19. What’s more, the gene correlation study comprehensively these three genes in IPF according to their potential functions, related pathways, efficacy values, and clinical applications. Our study provides a new angle to understanding the association between COVID-19 and IPF. The survival-related genes from COVID-19 explored in our study showed the potential to predict the prognosis of IPF patients and could be valuable as candidate biomarkers and potential targets for the therapy in IPF patients and IPF patients with COVID-19 in the future.

## Data Availability

Publicly available datasets were analyzed in this study. This data can be found here: http://www.ncbi.nlm.nih.gov/geo/.
